# Determinants of COVID-19 Vaccine Acceptance and Hesitancy: A Health Care Student-Based Online Survey in Northwest China

**DOI:** 10.3389/fpubh.2021.777565

**Published:** 2022-01-06

**Authors:** Juxia Zhang, Judith Dean, Yuhuan Yin, Dongping Wang, Yanqing Sun, Zhenhua Zhao, Jiancheng Wang

**Affiliations:** ^1^Clinical Educational Department, Gansu Provincial Hospital, Lanzhou, China; ^2^School of Public Health, The University of Queensland, Herston, QLD, Australia; ^3^School of Nursing, Gansu University of Chinese Medicine, Lanzhou, China; ^4^Department of Human Resource, Gansu Provincial Hospital, Lanzhou, China; ^5^Geriatrics Department, Gansu Provincial Hospital, Lanzhou, China

**Keywords:** COVID-19 vaccine, healthcare students, vaccine hesitant, vaccine acceptance, China

## Abstract

**Background:** With the spread of COVID-19 around the world, herd immunity through vaccination became a key measure to control the pandemic, but high uptake of vaccine is not guaranteed. Moreover, the actual acceptance of COVID-19 vaccination and associated factors remain uncertain among health care students in Northwest China.

**Methods:** A cross-sectional survey of a sample of 631 health care students was performed using a questionnaire developed through Wen Juan Xing survey platform to collect information regarding their attitudes, beliefs, and acceptance of COVID-19 vaccination. Binary logistic regression analyses were performed to identify the association between vaccination willingness and demographics, attitudes, and beliefs to determine the factors that actually effect acceptance and hesitancy of COVID-19 vaccine among health care students.

**Results:** Overall, 491 (77.81%) students actually received the COVID-19 vaccine, and of the 140 unvaccinated, 69 were hesitant and 71 rejected. Binary logistic regression analysis showed that the actually vaccinated individuals were those who mostly believed in the effectiveness of the COVID-19 vaccine (OR = 2.94, 95%CI: 1.37, 6.29), those who mostly felt it is their responsibility to receive the vaccine to protect others from infection (OR = 2.75, 95%CI: 1.45, 5.23), with less previous experience about other vaccines (OR = 1.70, 95%CI: 1.06, 2.72), students who mostly thought COVID-19 to be very severe (OR = 1.77, 95%CI: 1.07, 2.93), and students who mostly thought the COVID-19 vaccine was one of the best protection measures (OR = 1.68, 95%CI: 1.03, 2.76). Concerns about side effects of vaccines (OR = 0.30, 95%CI: 0.18, 0.51) and the use of personal protective behavior as an alternative to the COVID-19 vaccination (OR = 0.16, 95%CI: 0.06, 0.39) hindered the vaccine acceptance.

**Conclusions:** Our study showed higher COVID-19 vaccine acceptance among healthcare students. However, the individuals with vaccine hesitancy and rejection were still worrying. Vaccine safety and effectiveness issues continue to be a major factor affecting students' acceptance. To expand vaccine coverage in response to the COVID-19 pandemic, appropriate vaccination strategies and immunization programs are essential, especially for those with negative attitudes and beliefs.

## Introduction

Coronavirus disease 2019 (COVID-19) is still spreading around the world. Up to August 2021, a total of 216.30 million COVID-19 patients were confirmed worldwide with 4.49 million deaths (1). To mitigate the spread of COVID-19 and its impact, tireless measures have been taken. Of them, universal vaccination against COVID-19 is expected to be the most efficient preventive measure for limiting the pandemic (2). At the end of 2020, COVID-19 vaccines were approved for use. It has been reported that there have been more than 160 candidate vaccines to date, with around 20 candidates in clinical evaluation (3). With the availability of effective COVID-19 vaccines, one critical step is to achieve herd immunity through mass use of the vaccines in the general population (3). Health care workers (HCWs) including health care students are at much greater risk of developing COVID-19 with a 11-fold increased positive rates as compared to the general community (4). They also played an important role in transmission of nosocomial infections to people through the hands, equipment, and enclosing surfaces in the clinical settings (5). Therefore, HCWs including health care students were targeted in the first phase for vaccination across different countries (6, 7).

Vaccine hesitancy is defined by the World Health Organization (WHO) Strategic Advisory Group of Experts on Immunization (SAGE) as a “delay in acceptance or refusal of vaccination despite the availability of vaccination services” (8) which is recognized as a serious public health threat (9). The public would assume that given the nature of the profession, HCWs should not have hesitancy to accept the COVID-19 vaccine (10). However, evidence shows that HCWs can themselves be vaccine hesitant, which may further impact hesitancy and aversion to receiving the vaccine among the public (11, 12). During the COVID-19 pandemic, students in health care professions helped serve on the front line, worked as volunteers with vaccination campaigns, and took on roles where there could be greater exposure to COVID-19 infection (13). Additionally, health care students are prone to an increased risk of contracting infectious diseases as part of their clinical training requirement (13). Therefore, they are obliged to receive certain vaccines especially in the clinical years of their undergraduate courses. Different studies have been produced to fully understand the drivers of vaccine hesitancy and its prevalence among medical students. However, a global assessment including students/trainees of health care professions from 39 countries, conducted between April 2020 and April 2021, indicated that the overall rate of COVID-19 vaccination hesitancy was 18.9% (10). In a study conducted in the United States in February 2021, only 45% of nursing students intended to be vaccinated (14). In an Egyptian study conducted in January 2021, only 35% of the medical students accepted the vaccination (6). This issue poses a significant problem for ongoing efforts to control the current COVID-19 pandemic.

Since March 2020, China has reached a well-contained phase with work, study, and life of the general population returned to relatively normality (15). In addition, as the leading country in the development of COVID-19 vaccines, the China Health Authority approved its first COVID-19 inactivated vaccine on December 31, 2020, and the vaccines were firstly scheduled to use in groups with higher risk of infection aged 18–59 (e.g., workers in the cold-chain logistics sector, customs inspectors, health professionals, community workers) (16). A study conducted in May 2020 showed the COVID-19 vaccine hesitancy in Chinese general community was 20% (17), and meanwhile, HCWs showed more willingness to be vaccinated with 6.1% of hesitancy (18). In July 2021, the steady situation in China was dramatically overturned since the importation of the mutant delta viral strain from overseas. Due to management issues in Nanjing International Airport, workers within the airport were infected by the delta strain. Consequently, the imported delta strain has been transmitted rapidly to 27 provinces in China. Facing the possibility of outbreak resurgence, public enlightenment was of notable importance. For the global vaccination campaign to be effective, any reasons for hesitation about vaccination must be addressed. However, at present, few studies have investigated the prevalence of COVID-19 vaccine acceptance and hesitancy among health care students in Northwest China, which was less hit during the first COVID-19 wave compared to other Chinese regions; moreover, factors associated with vaccination motivation and hesitation are also unclear. Having this information could help educators identify students with hesitation and develop effective interventions to promote adherence to vaccination. In light of these gaps, our aim was to identify factors associated with the COVID-19 vaccine acceptance among health care students.

## Methods

### Ethics Statement

This study was approved by the Medical Ethics Committee at Gansu Provincial Hospital, Lanzhou, China. Respondents were informed that their participation was voluntary, and consent was implied on the completion of the questionnaire.

### Sample Size

According to a previous study, currently the average proportion of people who are vaccinated is 54.6% in China (17). Assume a proportion of vaccination acceptance of *p* = 50% and a precision level of 4% (50 ± 4%). The sample size was calculated as follows (19):


(1) $$\begin{array}{l} \frac{Z_{\alpha /2}^{2}\;\left(1-p\right)\;p}{\delta^{2}}\; \end{array}$$

where confidence level Z (α/2) = 1.96, and δ is the allowable error (0.04). The resulting sample size of 600 was increased of 10–12% to account for questionnaires discard due to lack of information and filling errors.

### Survey Design and Participants

We conducted a cross-sectional, web-based survey using an online questionnaire. The survey was administered from August 16–20, 2021, through the biggest online survey platform in China-Wen Juan Xing. Potential participants for the study were conveniently drawn from eight medical universities in Gansu, China, which were located in Northwest China, with total confirmed COVID-19 cases of 169. The eligible students were students enrolled, at any year level, in an undergraduate health care profession program majoring in a specialty area (nursing, clinical medicine, clinical laboratory, radiology, midwifery, traditional Chinese medicine, rehabilitation medicine, psychology, pharmaceutics). The director of students management of each included university was asked to randomly select 80 students. WeChat (the most popular social media platform in China) was used to distribute the survey link to the selected students *via* WeChat contact lists. The students were also asked to share the survey link with other health care students who were eligible and willing to take part in the study. Finally, the survey link was reached by 673 students from 13 schools or universities with all of them finishing the survey.

### Instruments

In the first part of the survey page, we described the nature of the study and set informed consent that the respondents can choose to agree or disagree to continue or stop response to the questionnaire. Basically, the questionnaire was designed based on previous studies to evaluate the vaccine acceptance among students (6, 14). A preliminary pilot experiment was conducted on 30 health care students who interned in Gansu Provincial Hospital with Cronbach's alpha coefficients of the questionnaire internal consistency reliability as 0.86, which means that the questionnaire can accurately measure the degree of vaccination behavior among health care students. The final questionnaire included the following information: (1) Demographics and health status: age, gender, education level, attending volunteer activity, history of COVID-19 training, previous history of other vaccines, the way of acquiring knowledge about vaccine (Television or journals, internet, advice from others), health status. (2) Attitudes and beliefs of COVID-19 vaccination: Awareness of the severity of COVID-19, thinking COVID-19 vaccine is one of the best protection measures, having a responsibility to be vaccinated to protect others, expectation to return to normal outdoor travel and activities, trust in the effectiveness of COVID-19 vaccine, thinking vaccination is a way to ensure one's safety, complying with health ministry recommendations, concerns about side effects of vaccines, concern about the ineffectiveness of vaccines, considering the personal protection behaviors as a substitute of vaccination in the prevention of COVID-19, absence of confirmed cases locally, presence of contraindications for COVID-19 vaccination. Each subject answered all the questions. (3) Acceptance of COVID-19 vaccine: measured using a one-item question (Currently vaccine against COVID-19 is available without payment, have you taken it?) on a four-point scale (“definitely no” to “definitely yes”) which stands for the actual accept group (definitely yes), hesitancy group (“probably yes” or “probably no”), and refusal group (definitely no). In the analysis, “definitely no,” “probably yes,” and “probably no” were all referred to as unvaccinated.

### Statistical Analysis

Statistical analysis was performed using SPSS 21.0, frequencies and proportions were used to describe the demographic characteristics, attitudes, beliefs, and behavior of COVID-19 vaccination. Chi-square test and Fisher's exact test were performed to preliminarily analyse various independent variables (i.e., demographics, vaccine history, vaccine knowledge, sources of information, attitudes and beliefs) related to each of the main outcomes (actual vaccination acceptance yes/no). The actual accept group and the non-vaccinated group including students who hesitated and refused were compared. Next, binary logistic analysis with the forward variable selection method was used to examine the independent factors related to COVID-19 vaccination behavior. The dependent variable was the vaccination acceptance (non-vaccinated = 0, actual vaccination = 1), with the significant factors in univariate analyses included as independent variables. In order to prevent over fitting of logistic regression model, we used Chi-square test and calculating the Phi correlation coefficient for each pair-wise correlation. Then we discarded from logistic regression model estimation those variables that have a correlation coefficient phi higher than 0.8. Significantly statistical difference was set at 0.05 (two-tailed). The odds ratio (OR), standard error (SE), 95% confidence interval (CI), and *p*-values were reported. As the students from each school out of the previously selected eight were < 10, we did not further analyze the differences of geographic location of 13 medical schools with respect to the burden of COVID-19 experienced.

## Results

### Students' Characteristics

After sorting out the 673 collected questionnaires, we excluded 42 invalid questionnaires (lack of information, filling errors) and finally 631 valid questionnaires were included with a rate of 93.76%. Among 631 respondents, 79.71% were female, the mean age was 20.08 ± 1.7 years with 61.17% aged below 22, and 91.13% were students from universities in Gansu Province. More than half of the students (56.89%) have attended clinical practice, 34.55% were students majoring in nursing, and the majority (75.12%) of the students have received training on COVID-19. In total, 61.33% of students had no prior experience with other vaccines, and 1.43% thought they had presence of any disease that might affect vaccination, including epilepsy, history of severe anaphylactic shock, and congenital heart disease (Table 1).

**Table 1:** Demographic characteristics of health care students.

**Characteristics**	**Overall respondents**	**Actual vaccination^**a**^**	**Non-vaccinated^**b**^**	**X^**2**^**	***P***
	**(*N* = 631), *n* (%)**	**(*N* = 491), *n* (%)**	**(*N* = 140), *n* (%)**		
Gender				3.28	0.08
Female	503 (79.71)	399 (81.26)	104 (74.29)		
Male	128 (20.29)	92 (18.74)	36 (25.71)		
Age group					
<22	386 (61.17)	311 (63.34)	75 (53.57)	4.38	**0.04**
≥22	245 (38.83)	180 (36.66)	65 (46.43)		
Profession				2.16	0.12
Clinical medicine	75 (11.89)	56 (11.41)	19 (13.57)		
Nursing	218 (34.55)	170 (34.62)	48 (34.29)		
Pharmacy	99 (15.69)	81 (16.50)	18 (12.86)		
Medical technician	78 (12.36)	70 (14.26)	8 (5.71)		
Others	161 (25.52)	114 (23.22)	47 (33.57)		
Educational level				4.09	**0.04**
Junior college student	318 (50.40)	258 (52.55)	60 (42.86)		
Undergraduate or graduate student	313 (49.60)	233 (47.45)	80 (57.14)		
The geographical location of the school				2.16	0.12
Gansu Province	575 (91.13)	459 (93.48)	116 (82.86)		
Other Provinces	56 (8.87)	32 (6.52)	24 (17.14)		
Clinical practice				0.71	0.44
No	272 (43.11)	216 (43.99)	56 (40.00)		
Yes	359 (56.89)	275 (56.01)	84 (60.00)		
Volunteered to COVID-19				0.19	0.67
No	451 (71.47)	353 (71.89)	98 (70.00)		
Yes	180 (28.53)	138 (28.11)	42 (30.00)		
Received training on COVID-19 prevention	1.31	0.27
No	157 (24.88)	117 (23.83)	40 (7.14)		
Yes	474 (75.12)	374 (76.17)	100 (71.43)		
Acceptance of other vaccines				7.44	**0.01**
No	387 (61.33)	315 (64.15)	72 (51.43)		
Yes	244 (38.67)	176 (35.85)	68 (48.57)		
Information about the COVID-19 vaccine					
Television or journals	179 (28.37)	144 (29.33)	35 (25.00)	1.05	0.60
Internet	386 (61.17)	297 (60.49)	89 (63.57)		
Advise from others	66 (10.46)	50 (10.18)	16 (11.43)		
Presence of any disease				/	0.42^#^
No	622 (98.57)	485 (98.78)	137 (97.86)		
Yes	9 (1.43)	6 (1.22)	3 (2.14)		

a *Actual vaccination = willing and have completed vaccination*;

b *Non-vaccinated = vaccine hesitation+vaccine resistance; Other vaccines = influenza vaccine, hepatitis B vaccine, and HPV vaccine; Presence of any disease (meaning students thought they had any diseases that might affect vaccination) = epilepsy, history of severe anaphylactic shock, and congenital heart disease*.

# *p-value for Fisher's exact test*.

*Bold values show significant differences*.

### Attitudes and Beliefs of COVID-19 Vaccination

Of the 631 students included, most (75.91%) thought that COVID-19 was very serious, and 58.64% believed that vaccine was one of the best protection measures against COVID-19. In total, 88.36% of them thought they had responsibility to receive the vaccine to protect others, 96.67% wanted to resume normal outdoor travel and activities, and most (90.17%) believed the effectiveness of the COVID-19 vaccine. In addition, 59.11% wanted to comply with health ministry recommendations, while 58.64% of students were concerned about the side effects of vaccines, and 10.78% considered personal protective behavior as an alternative to vaccination against COVID-19 (Table 2).

**Table 2:** Participants' attitudes and beliefs toward COVID-19 vaccine.

**Attitudes and beliefs**	**Overall respondents**	**Actual vaccination^**a**^**	**Non-vaccinated^**b**^**	**X^**2**^**	***P***
	**(*N* = 631), *n* (%)**	**(*N* = 491), *n* (%)**	**(*N* = 140), *n* (%)**		
The severity of COVID-19				13.30	**<0.01**
Generally serious	152 (24.09)	102 (20.77)	50 (35.71)		
Very serious	479 (75.91)	389 (79.23)	90 (64.29)		
COVID-19 vaccine is one of the best protection measures				34.27	**<0.01**
No	261 (41.36)	173 (35.23)	88 (62.86)		
Yes	370 (58.64)	318 (64.77)	52 (37.14)		
Having a responsibility to be vaccinated to protect others				79.70	**<0.01**
No	105 (16.64)	47 (9.57)	58 (41.43)		
Yes	526 (83.36)	444 (90.43)	82 (58.57)		
Expectation to return to normal outdoor travel and activities				/	**<0.01** ^#^
No	21 (3.33)	8 (1.63)	13 (9.29)		
Yes	610 (96.67)	483 (98.37)	127 (90.71)		
Trust in the effectiveness of COVID-19 vaccine				55.98	**<0.01**
Little trust	62 (9.83)	25 (5.09)	37 (26.43)		
Great trust	569 (90.17)	466 (94.91)	103 (73.57)		
Thinking vaccination is a way to ensure one's safety				0.00	1.00
No	409 (64.82)	318 (64.77)	91 (65.00)		
Yes	222 (35.18)	173 (35.23)	49 (35.00)		
Complying with health ministry recommendations				10.02	**<0.01**
No	258 (40.89)	217 (44.20)	41 (29.29)		
Yes	373 (59.11)	274 (55.80)	99 (70.71)		
Concerns about side effects of vaccines				36.16	**<0.01**
No	261 (41.36)	234 (47.66)	27 (19.29)		
Yes	370 (58.64)	257 (52.34)	113 (80.71)		
Concerns about the ineffectiveness of vaccines				27.14	**<0.01**
No	578 (91.60)	465 (94.70)	113 (80.71)		
Yes	53 (8.40)	26 (5.30)	27 (19.29)		
Considering the personal protection behaviors as a substitute of vaccination in the prevention of COVID-19				35.93	**<0.01**
No	563 (89.22)	475 (96.74)	88 (62.86)		
Yes	68 (10.78)	16 (3.26)	52 (37.14)		
Absence of confirmed cases locally				/	**<0.01** ^#^
No	609 (96.51)	482 (98.17)	127 (90.71)		
Yes	22 (3.49)	9 (1.83)	13 (9.29)		
Presence of contraindications for COVID-19 vaccination				/	**<0.01** ^#^
No	614 (97.31)	486 (98.98)	128 (91.43)		
Yes	17 (2.69)	5 (1.02)	12 (8.57)		

a *Actual vaccination = willing and have completed vaccination*;

b *non-vaccinated = vaccine hesitation+vaccine resistance; Generally serious = COVID-19 is of low risk and is not a threatening disease; Very serious = COVID-19 is very risky and life-threatening*.

# *p-value for Fisher's exact test*.

*Bold values show significant differences*.

### Factors Associated With COVID-19 Vaccination Behavior

**Figure 1:**
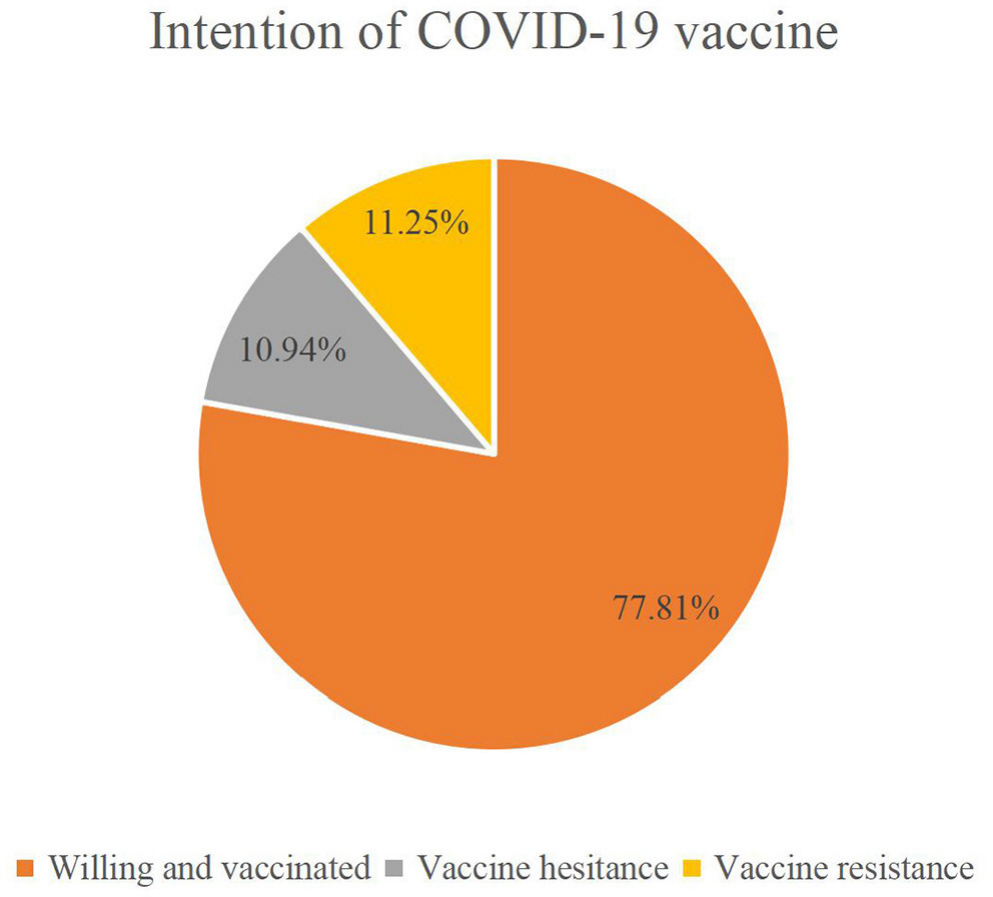
Willingness to receive the COVID-19 vaccine.

Overall, 491 (77.81%) students actually received the COVID-19 vaccine, and of the 140 unvaccinated, 69 were hesitant and 71 rejected (Figure 1).

In order to eliminate the influence of confounding factors on motivations of COVID-19 vaccination, 14 statistically significant factors from the univariate analysis in Tables 1, 2 were entered into a binary regression model. Multivariate analysis results in Table 3 showed that the greatest factor affecting vaccine acceptance was trust in the effectiveness of COVID-19 vaccine (OR = 2.94, 95%CI: 1.37, 6.29) and feeling the COVID-19 vaccine as a responsibility to protect others (OR = 2.75, 95% CI: 1.45, 5.23). The actually vaccinated individuals were also more likely to be students who had not received other vaccines (OR = 1.70, 95%CI: 1.06, 2.72), students who thought COVID-19 to be very severe (OR = 1.77, 95%CI: 1.07, 2.93), students who thought COVID-19 vaccine was one of the best protection measures (OR = 1.68, 95%CI: 1.03, 2.76), and students who felt it was their responsibility to receive the vaccine to protect others from infection (OR = 2.75, 95%CI: 1.45, 5.23).

**Table 3:** Factors associated with acceptance of COVID-19 vaccine.

**Factors**	**B**	**S.E**	**Wals**	***P***	**OR (95%CI)**
Age group (≥22)	−0.24	0.25	0.90	0.35	0.79 (0.48, 1.29)
Educational level (undergraduate or graduate student)	−0.22	0.26	0.74	0.39	0.80 (0.48, 1.33)
Acceptance of other vaccines previously (no)	0.53	0.24	4.83	**0.04**	1.70 (1.07, 2.93)
The severity of COVID-19 (very serious)	0.57	0.26	5.00	**0.03**	1.77 (1.07. 2.93)
COVID-19 vaccine is one of the best protection measures (yes)	0.52	0.25	4.28	**0.04**	1.68 (1.03, 2.76)
Having a responsibility to be vaccinated to protect others (yes)	1.01	0.33	9.56	**<0.01**	2.75 (1.45, 5.23)
To return to normal outdoor travel and activities (yes)	0.83	0.57	2.15	0.14	2.30 (0.76, 7.27)
Trust in the effectiveness of COVID-19 vaccine (yes)	1.08	0.39	7.71	**<0.01**	2.94 (1.37, 6.29)
To comply with health ministry recommendation (yes)	−0.37	0.25	2.24	0.13	0.69 (0.43, 1.12)
Concerns about the ineffectiveness of vaccines (no)	0.63	0.39	2.63	0.11	1.87 (0.88, 3.99)
Concerns about side effects of vaccines (yes)	−1.20	0.27	19.46	**<0.01**	0.30 (0.18, 0.51)
Consider the personal protection behaviors as a substitute of vaccination in the prevention of COVID-19 (yes)	−1.84	0.46	15.99	**<0.01**	0.16 (0.06, 0.39)
There are no confirmed cases locally (yes)	−0.89	0.61	2.09	0.15	0.41 (0.12, 1.37)
They have contraindications for COVID-19 vaccination (yes)	−0.92	0.61	2.29	0.13	0.40 (0.12, 1.31)

*Dependent variable assignment, unvaccinated =0, actual vaccinated =1; OR, odds ratio; CI, confidence interval; P < 0.05 was considered to indicate significance; Bold values show significant differences*.

Concerns about side effects of vaccines (OR = 0.30, 95%CI: 0.18, 0.51) and the use of personal protective behavior as an alternative to COVID-19 vaccination (OR = 0.16, 95%CI: 0.06, 0.39) hindered the vaccine acceptance.

## Discussion

In this study, we surveyed the acceptance of COVID-19 vaccine and related factors among health care students. Previous surveys among students majoring in nursing, dental, and medical showed the vaccine willingness rates varying from 56 to 91.9% in different countries or regions (6, 10, 13, 20–23). In the present study, the number of students willing to be vaccinated reached 88.75% with 77.81% of them actually vaccinated. Similar results were also found in Chinese nursing students (84.4%) during February–April 2021 (24), as well as Chinese HCWs with 86.2% acceptance during January 2021 (18). These high acceptance rates of COVID-19 vaccination may be associated with higher literacy on health-related issues, as well as the Chinese government's policy to complete the COVID-19 vaccination in all HCWs (25).

Importantly, multivariable analysis found that the factor that explained the greatest proportion of the variance in vaccination acceptance was trust in the effectiveness of the COVID-19 vaccine, and feeling the COVID-19 vaccine as a responsibility to protect others, similar results were also found in medical students (26, 27) and the general population (28). In our study, the majority of students (90.17%) believed the efficacy of vaccines, yet a few students were skeptical. Previous studies found that health care workers who believe in vaccines were more likely to recommend vaccines to friends, families, and their patients (18, 29). This suggested that people's general positive sense is driving vaccination intention at this point, and interventions targeted at modifying such health beliefs about COVID-19 may lead to improved vaccination rates. Thus, it is necessary for school and hospital leaders to promote widespread trust in vaccines among students through active role models and the mobilization of influencers within institutions. Students who felt they had a responsibility to get vaccinated to protect others from infection were more likely to get vaccinated. The more people have doubts about COVID-19 vaccination, the more courage and responsibility should be taken to break the doubts in society (18). Health care students were actively taking the lead in vaccination and making contributions to the country's fight against the epidemic, which reflected not only support for the epidemic prevention and value for the achievements of the fight against the epidemic but also a high level of personal ethics and trust in the health system (17, 25). The COVID-19 pandemic and its associated social isolation have worsened the mental state of people (26), and positive social outcomes such as an end to social isolation or attending in-person classes may be an important driver for increased uptake of vaccine in this population (30). Therefore, schools should actively carry out educational lectures to establish students' good sense of responsibility so that more health care students can take the initiative to shoulder the responsibility, play the vanguard role of youth, and take the lead in voluntary vaccination.

We also found that students who think COVID-19 is very serious and those think COVID-19 vaccine is one of the best protection measures were more likely to get vaccinated, which was similar to other studies (12, 18). Most of the health care students had high perceptions of the severity of COVID-19 and perceived benefits in obtaining the vaccine. However, zero confirmed delta strain cases reported in Gansu during the study period lead to insufficient awareness and risk of COVID-19 among some health care students. There is also evidence that as the number of confirmed cases continues to increase, people may be more aware of the severity of COVID-19, which further increase COVID-19 vaccine acceptance (31). Bai et al.'s research showed that students who attend health-related courses have a higher awareness of the risk of COVID-19 and thus higher vaccination rates (32). Our study did not find a significant effect of this factor on vaccination behavior, but it is worth noting that a quarter of the students in our study had still not been trained on COVID-19, which also reflects the importance that the government and schools attached to the prevention and controlling of COVID-19. Wong et al.'s study found that dissemination of knowledge about the severity of COVID-19 is needed as it has been found to be useful in influencing vaccine-related behavior (33).

In our sample there were 22% of hesitant students or students who rejected the COVID-19 vaccination. Similar results were found in India (13) and Poland (27). Even higher vaccine hesitancy was reported by medical students in Uganda (62.7%) (20), USA (44.5%) (22), and Egypt (19.4%) (6). The occurrence of COVID-19 vaccination hesitancy and refusal in students of health care professions is concerning. Without COVID-19 vaccination, health care students themselves can pose a risk of infection in high burden situations. Given that individuals are more likely to accept COVID-19 vaccination with the health care providers' recommendation, if acceptance of COVID-19 vaccines remains lower in health care professionals, how will they be able to make recommendations to others (10)?

Our results found that the most reported barrier for the vaccination among unvaccinated individuals was fear of adverse effects. Lack of vaccine confidence, in particular mistrust in the safety of a COVID-19 vaccine, represents a significant barrier to vaccine acceptance not only in our study but also indicated in dental students (34), medical students (6), as well as among HCWs (35, 36) and community population (26), which could significantly reduce an individual's confidence in the vaccine or select to delay vaccination (37–43). Thus, more information about vaccine safety, efficacy, and side effects should be addressed in a transparent way by health education campaigns. In particular, health care professionals and school leaders should be encouraged to share their COVID-19 vaccination experiences with friends or relatives to build vaccine confidence and trust (44). In addition, in our sample, the students who had not received other vaccines previously were more likely to engage in COVID-19 vaccination behavior, which was not surprising as the adverse reactions to other vaccinations in young people has been repeatedly exaggerated in the news, stoking public doubts about the vaccine's safety, which are an important reason for an individual's hesitation about vaccines (43). Universal vaccination is an important measure to control the epidemic, especially as high vaccination coverage among younger adults could be key to reaching adequate herd immunity. Strategies are required to reach individuals who distrust information from their government and health officials with accurate information concerning risks and beliefs about vaccine immunity. Future studies may benefit from qualitative methods to identify the factors that contribute to the perceived fears of vaccines, which likely include perceptions of the risk of side effect and the effectiveness against illness and associated benefits.

Similarly, our results also showed that students who consider personal protection behaviors as a substitute of vaccination in the prevention of COVID-19 were more likely to hesitate and reject vaccination, which reflected the lack of medical knowledge about COVID-19 vaccine among health care students. Some studies showed that this might be because of the misinformation dissemination within some media (36, 45). This requires the government to crack down disinformation and misinformation, and provide the public with the most authoritative information in a timely manner (45). Thus, developing an educational framework for governments and health care providers to inform the public about the risks of delaying or refusing vaccination is critical to mitigate the harmful effects of the COVID-19 pandemic (46). Meanwhile, to improve COVID-19 vaccination behavior among health care students, schools need to conduct more educational and social campaigns describing the importance of vaccination.

### Strength and Limitation

To our knowledge, there was limited research regarding health care students' actual acceptance of COVID-19 vaccination. This study provides information on the impact of the COVID-19 pandemic on acceptance of vaccination among this young group, and identify factors associated with acceptance of COVID-19 vaccination.

There are limitations to this study. First, due to the selection method, the sample cannot be considered as representative of areas other than Gansu province. Moreover, the voluntary effect, i.e., participants' self-selection, does not allow us to consider the prevalence of vaccine acceptance generalizable to the health care student population. Despite these limitations, these findings are not inconsistent with the findings from previous studies about health care students' vaccine hesitancy. In the future, we need to select a large sample data to analyze whether there are differences in students' willingness to vaccinate in different areas. Second, despite our study recruiting an adequate number of students, the study focused on the working setting, and thus years of participants studying in university were not collected. In addition, this paper did not compare the intention of vaccination during or after the pandemic, and future study should compare this difference in detail.

## Conclusions

In the present survey, the majority of students already received the COVID-19 vaccine. However, those who hesitated or rejected the vaccine were still worrying. Vaccine safety and effectiveness issues continue to be a major factor affecting students' acceptance. The negative attitudes and beliefs about vaccinations toward COVID-19 were highly correlated with vaccination hesitancy or rejection. It is important to design an evidence-based strategy to promote the uptake of vaccination for health care students. Additionally, effectiveness and the safety of the vaccine will be important when rolling out the COVID-19 vaccine. Future studies are needed to explore reasons of COVID-19 vaccination acceptance, and to examine the effectiveness of promotion strategies.

## Data Availability Statement

The original contributions presented in the study are included in the article/supplementary material, further inquiries can be directed to the corresponding author.

## Ethics Statement

The studies involving human participants were reviewed and approved by Medical Ethics Committee at Gansu Provincial Hospital. The patients/participants provided their written informed consent to participate in this study.

## Author Contributions

JZ, JD, YS, and JW: conception and design of the study. JZ and DW: acquisition of data. YY, JD, and JW: analysis and interpretation of data. JZ and YY: drafting the article. ZZ, JD, YY, JZ, and JW: revising the article. JW and JZ: funding acquisition. All authors contributed to the final approval of the version to be submitted.

## Funding

This research was funded by Natural Science Foundation of Gansu Province (Grant number 21JR7RA613), Lanzhou Chengguan District Science and Technology plan project (Grant number 2019RCCX0011), Horizontal project of Lanzhou Hand-foot Surgery Hospital (Grant number 071100278), and Natural Science Foundation of Gansu Province (Grant number 21JR7RA607).

## Conflict of Interest

The authors declare that the research was conducted in the absence of any commercial or financial relationships that could be construed as a potential conflict of interest.

## Publisher's Note

All claims expressed in this article are solely those of the authors and do not necessarily represent those of their affiliated organizations, or those of the publisher, the editors and the reviewers. Any product that may be evaluated in this article, or claim that may be made by its manufacturer, is not guaranteed or endorsed by the publisher.

## References

[1] WHO. Coronavirus Disease (?COVID-19)?: Weekly Epidemiological Update on COVID-19 (2021). Available online at: https://www.who.int/publications/m/item/weekly-epidemiological-update-on-covid-19 (accessed 24 August, 2021)

[2] Malik AA McFadden SM Elharake J Omer SB. Determinants of COVID-19 vaccine acceptance in the US. EClinicalMedicine. (2020) 26:100495. 10.1016/j.eclinm.2020.100495 32838242 PMC7423333

[3] Lurie N Saville M Hatchett R Halton J. Developing Covid-19 vaccines at pandemic speed. N Engl J Med. (2020) 382:1969–73. 10.1056/NEJMp2005630 32227757

[4] Nguyen LH Drew DA Graham MS Joshi AD Guo CG Ma W. Risk of COVID-19 among front-line health-care workers and the general community: a prospective cohort study. Lancet Public Health. (2020) 5:e475–83. 10.1016/S2468-2667(20)30164-X 32745512 PMC7491202

[5] Lake MA. What we know so far: COVID-19 current clinical knowledge and research. Clin Med. (2020) 20:124–7. 10.7861/clinmed.2019-coron 32139372 PMC7081812

[6] Saied SM Saied EM Kabbash IA Abdo SAE. Vaccine hesitancy: Beliefs and barriers associated with COVID-19 vaccination among Egyptian medical students. J Med Virol. (2021) 93:4280–91. 10.1002/jmv.26910 33644891 PMC8013865

[7] Eibensteiner F Ritschl V Nawaz FA Fazel SS Tsagkaris C Kulnik ST. People's willingness to vaccinate against COVID-19 despite their safety concerns: twitter poll analysis. J Med Internet Res. (2021) 23:e28973. 10.2196/28973 33872185 PMC8086789

[8] MacDonald NE. SAGE Working Group on Vaccine Hesitancy. Vaccine hesitancy: definition, scope and determinants. Vaccine. (2015) 33:4161–4. 10.1016/j.vaccine.2015.04.036 25896383

[9] Ebrahimi OV Johnson MS Ebling S Amundsen OM Halsøy Ø Hoffart A. Risk, trust, and flawed assumptions: vaccine hesitancy during the COVID-19 pandemic. Front Public Health. (2021) 9:700213. 10.3389/fpubh.2021.700213 34277557 PMC8281037

[10] Mustapha T Khubchandani J Biswas N. COVID-19 vaccination hesitancy in students and trainees of healthcare professions: a global assessment and call for action. Brain Behav Immun Health. (2021) 16:100289. 10.1016/j.bbih.2021.100289 34226892 PMC8241692

[11] Qattan AMN Alshareef N Alsharqi O Al Rahahleh N Chirwa GC Al-Hanawi MK. Acceptability of a COVID-19 vaccine among healthcare workers in the Kingdom of Saudi Arabia. Front Med. (2021) 8:644300. 10.3389/fmed.2021.644300 33732723 PMC7959705

[12] Li M Luo Y Watson R Zheng Y Ren J Tang J. Healthcare workers' (HCWs) attitudes and related factors towards COVID-19 vaccination: a rapid systematic review. Postgrad Med J. (2021) 30:postgradmedj-2021-140195. 10.1136/postgradmedj-2021-140195 37319159

[13] Jain J Saurabh S Kumar P Verma MK Goel AD Gupta MK. COVID-19 vaccine hesitancy among medical students in India. Epidemiol Infect. (2021) 149:e132. 10.1017/S0950268821001205 34011421 PMC8185413

[14] Kregar Velikonja N Dobrowolska B Stanisavljević S Erjavec K Globevnik Velikonja V Verdenik I. Attitudes of nursing students towards vaccination and other preventive measures for limitation of COVID-19 pandemic: cross- sectional study in three European Countries. Healthcare. (2021) 9:781. 10.3390/healthcare9070781 34206217 PMC8305964

[15] Wang J Jing R Lai X Zhang H Lyu Y Knoll MD. Acceptance of COVID-19 vaccination during the COVID-19 pandemic in China. Vaccines. (2020) 8:482. 10.3390/vaccines8030482 32867224 PMC7565574

[16] *Press Conference of the Joint Prevention and Control Mechanism of the State Council (2021)*. Available online at: http://www.gov.cn/xinwen/gwylflkjz145/index.htm. (accessed January 14, 2021)

[17] Lin Y Hu Z Zhao Q Alias H Danaee M Wong LP. Understanding COVID-19 vaccine demand and hesitancy: a nationwide online survey in China. PLoS Negl Trop Dis. (2020) 14:e0008961. 10.1371/journal.pntd.0008961 33332359 PMC7775119

[18] Wang MW Wen W Wang N Zhou MY Wang CY Ni J. COVID-19 vaccination acceptance among healthcare workers and non-healthcare workers in China: a survey. Front Public Health. (2021) 9:709056. 10.3389/fpubh.2021.709056 34409011 PMC8364953

[19] Schmidt SAJ Lo S Hollestein LM. Research techniques made simple: sample size estimation and power calculation. J Invest Dermatol. (2018) 138:1678–82. 10.1016/j.jid.2018.06.165 30032783

[20] Kanyike AM Olum R Kajjimu J Ojilong D Akech GM Nassozi DR. Acceptance of the coronavirus disease-2019 vaccine among medical students in Uganda. Trop Med Health. (2021) 49:37. 10.1186/s41182-021-00331-1 33985592 PMC8116637

[21] Szmyd B Bartoszek A Karuga FF Staniecka K Błaszczyk M Radek M. Medical students and SARS-CoV-2 vaccination: attitude and behaviors. Vaccines. (2021) 9:128. 10.3390/vaccines9020128 33562872 PMC7915119

[22] Mascarenhas AK Lucia VC Kelekar A Afonso NM. Dental students' attitudes and hesitancy toward COVID-19 vaccine. J Dent Educ. (2021) 85:1504–10. 10.1002/jdd.12632 33913152 PMC8242421

[23] Kelekar AK Lucia VC Afonso NM Mascarenhas AK. COVID-19 vaccine acceptance and hesitancy among dental and medical students. J Am Dent Assoc. (2021)152:596–603. 10.1016/j.adaj.2021.03.006 34030867 PMC7997309

[24] Jiang N Wei B Lin H Wang Y Chai S Liu W. Nursing students' attitudes, knowledge and willingness of to receive the coronavirus disease vaccine: a cross-sectional study. Nurse Educ Pract. (2021) 55:103148. 10.1016/j.nepr.2021.103148 34311170 PMC8275930

[25] Xu B Gao X Zhang X Hu Y Yang H Zhou YH. Real-world acceptance of COVID-19 vaccines among healthcare workers in perinatal medicine in China. Vaccines. (2021) 9:704. 10.3390/vaccines9070704 34199143 PMC8310137

[26] Shih SF Wagner AL Masters NB Prosser LA Lu Y Zikmund-Fisher BJ. Vaccine hesitancy and rejection of a vaccine for the novel coronavirus in the United States. Front Immunol. (2021) 12:558270. 10.3389/fimmu.2021.558270 34194418 PMC8236639

[27] Grochowska M Ratajczak A Zdunek G Adamiec A Waszkiewicz P Feleszko W. Comparison of the level of acceptance and hesitancy towards the influenza vaccine and the forthcoming COVID-19 vaccine in the medical community. Vaccines. (2021) 9:475. 10.3390/vaccines9050475 34066790 PMC8150871

[28] Khubchandani J Sharma S Price JH Wiblishauser MJ Sharma M Webb FJ. COVID-19 Vaccination hesitancy in the United States: a rapid national assessment. J Community Health. (2021) 46:270–7. 10.1007/s10900-020-00958-x 33389421 PMC7778842

[29] Wang K Wong ELY Ho KF Cheung AWL Chan EYY Yeoh EK. Intention of nurses to accept coronavirus disease 2019 vaccination and change of intention to accept seasonal influenza vaccination during the coronavirus disease 2019 pandemic: a cross-sectional survey. Vaccine. (2020) 38:7049–56. 10.1016/j.vaccine.2020.09.021 32980199 PMC7834255

[30] Kecojevic A Basch CH Sullivan M Chen YT Davi NK. COVID-19 Vaccination and intention to vaccinate among a sample of college students in New Jersey. J Community Health. (2021) 27:1–10. 10.1007/s10900-021-00992-3 33905034 PMC8077859

[31] McCord M. Coronavirus Vaccine: How Soon Will We Have One? (2020) Available online at: https://www.weforum.org/agenda/2020/03/vaccine-covid-19-coronavirus-pandemic-healthcare/ (accessed March 26, 2020)

[32] Bai W Cai H Liu S Liu H Qi H Chen X. Attitudes toward COVID-19 vaccines in Chinese college students. Int J Biol Sci. (2021) 17:1469–75. 10.7150/ijbs.58835 33907510 PMC8071773

[33] Wong LP Alias H Wong PF Lee HY AbuBakar S. The use of the health belief model to assess predictors of intent to receive the COVID-19 vaccine and willingness to pay. Hum Vaccin Immunother. (2020) 16:2204–14. 10.1080/21645515.2020.1790279 32730103 PMC7553708

[34] Riad A Abdulqader H Morgado M Domnori S Koščík M Mendes JJ. Global prevalence and drivers of dental students' COVID-19 vaccine hesitancy. Vaccines. (2021) 9:566. 10.3390/vaccines9060566 34072500 PMC8226539

[35] Wang J Feng Y Hou Z Lu Y Chen H Ouyang L. Willingness to receive SARS-CoV-2 vaccine among healthcare workers in public institutions of Zhejiang Province, China. Hum Vaccin Immunother. (2021) 17:2926–33. 10.1080/21645515.2021.1909328 33848217 PMC8381814

[36] Karafillakis E Dinca I Apfel F Cecconi S Wurz A Takacs J. Vaccine hesitancy among healthcare workers in Europe: a qualitative study. Vaccine. (2016) 34:5013–20. 10.1016/j.vaccine.2016.08.029 27576074

[37] Lin C Tu P Beitsch LM. Confidence and receptivity for COVID-19 vaccines: a rapid systematic review. Vaccines. (2020) 9:16. 10.3390/vaccines9010016 33396832 PMC7823859

[38] Peretti-Watel P Verger P Raude J Constant A Gautier A Jestin C. Dramatic change in public attitudes towards vaccination during the 2009 influenza A(H1N1) pandemic in France. Euro Surveill. (2013) 18:20623. 10.2807/1560-7917.es2013.18.44.20623 24176658

[39] Fisher KA Bloomstone SJ Walder J Crawford S Fouayzi H. Mazor KM. Attitudes toward a potential SARS-CoV-2 vaccine : a survey of US adults. Ann Intern Med. (2020) 173:964–73. 10.7326/M20-3569 32886525 PMC7505019

[40] Palamenghi L Barello S Boccia S Graffigna G. Mistrust in biomedical research and vaccine hesitancy: the forefront challenge in the battle against COVID-19 in Italy. Eur J Epidemiol. (2020) 35:785–8. 10.1007/s10654- 020-00675-8 32808095 PMC7431109

[41] Laurence D Görlich Y Simmenroth A. How do applicants, students and physicians think about the feminisation of medicine*?*- a questionnaire-survey. BMC Med Educ. (2020) 20:48. 10.1186/s12909-020-1959-2 32046693 PMC7014700

[42] Shaw J Stewart T Anderson KB Hanley S Thomas SJ Salmon DA. Assessment of U.S. health care personnel (HCP) attitudes towards COVID-19 vaccination in a large university health care system. Clin Infect Dis. (2021) 25:ciab054. 10.1093/cid/ciab054 33491049 PMC7929026

[43] Yoda T Katsuyama H. Willingness to receive COVID-19 vaccination in Japan. Vaccines. (2021) 9:48. 10.3390/vaccines9010048 33466675 PMC7828811

[44] Schaffer DeRoo S Pudalov NJ Fu LY. Planning for a COVID-19 vaccination program. JAMA. (2020) 323:2458–9. 10.1001/jama.2020.8711 32421155

[45] Wake AD. The willingness to receive COVID-19 vaccine and its associated factors: “vaccination refusal could prolong the war of this pandemic” - A systematic review. Risk Manag Healthc Policy. (2021) 14:2609–23. 10.2147/RMHP.S311074 34188572 PMC8232962

[46] García LY Cerda AA. Acceptance of a COVID-19 vaccine: a multifactorial consideration. Vaccine. (2020) 38:7587. 10.1016/j.vaccine.2020.10.026 33121656 PMC7588176

